# 

**Published:** 1901-03

**Authors:** 


### Society Notes.

### Texas State Medical Association.

The thirty-third annual meeting of the Texas State Medical Association will be held at Galveston, beginning on Tuesday, April 23rd (prox.), and continuing four days. The President, Dr. B. E. Hadra, Dallas, has issued the usual call and invitation to the physicians of Texas to be present. A delightful reunion is anticipated, and it is hoped, and expected, that there will be a larger attendance than usual. The occasion will afford an opportunity for a love feast among the brethren—a rejoicing over the victory of having at last secured the much needed medical legislation regarding the practice of medicine, to hold an interesting session, and to enjoy the social functions usually tendered the doctors on such occasions:—the hospitality of the generous and delightful people; to see the Island city in its *renaissance*—a veritable Phœnix, risen out of its ashes, and again blossoming like a rose; to see some vestiges which are still left of the historic wreck, and to have a good time generally. All work and no play makes Jack a dull boy. Put away the shovel and the hoe, take up the fiddle and the bow—so to say—and treat yourself to a little much needed relaxation. There are already too many dull boys in the ranks.

### Annual Meeting of the Association of Medical Officers of the Confederacy.

The annual meeting of this Association will be held in Memphis, Tenn., in conjunction with the annual reunion of the United Confederate Veterans, May 28th, 30th, 1901.

The Committee of Arrangements have sent out the following circular letter:

MEMPHIS, TENN., March 1, 1901.

DEAR DOCTOR: The Association of Medical Officers of the Army and Navy of the Confederacy will convene in Memphis, Tenn., May 28-30, 1901, during the meeting of the Confederate Reunion. All surgeons, assistant surgeons, acting assistant surgeons, or contract physicians and hospital stewards, in the army and navy of the Confederate States, and all regular physicians who are sons of Confederate veterans, are eligible to membership.

You are cordially invited to attend said meeting and contribute reports of important cases coming under your observation, and any reminiscenses worthy of preservation connected with your service in the army or navy of the Confederacy.

If you desire to become a member of the Association, and expect to attend the meeting next May, please fill out the enclosed blank and return same to the secretary at once, in order that your name may appear on the roll.

Respectfully,
G. B. MALONE, M. D., Chairman,
281 Main St., Memphis, Tenn.
A. L. ELCAN, M. D., Secretary,
Southern Express Building, Memphis, Tenn.

The enclosed blank alluded to contains space for name in full; time and place of enlistment; rank at time of enlistment; rank at close of war; character of service—army or navy; when and where surrendered; present address, and remarks.

Any further information desired will be most cheerfully furnished by Drs. Malone or Elcan, of Memphis, or Dr. Deering J. Roberts, Secretary of the Association, of Nashville, Tenn.

Nothing will be left undone to provide for the comfort, enjoyment and pleasure of the survivors of the late war between the States. Every man, woman and child in Memphis is fully enthused and thoroughly aroused, with a full determination that the occasion shall be both eventful and momentous to everyone who is so fortunate to attend.

The doctors of Memphis will see that their end of the line is fully kept up; and with a uniform railroad rate of one cent per mile over all Southern and Southeastern roads, the attendance should surely be a feature of the occasion.

